# Experiences of Young People with ADHD Transitioning from Child to Adult Health and Social Care Services in the UK: A Qualitative Systematic Review and Thematic Synthesis

**DOI:** 10.1192/bjo.2026.11116

**Published:** 2026-06-30

**Authors:** Iyinoluwa Popoola, Linn Johanna Gabbert

**Affiliations:** 1 King’s College London, London, United Kingdom; 2 Queen Mary University, London, United Kingdom

## Abstract

**Aims:**

To synthesise qualitative evidence on the lived experiences transitioning from child to adult health and social care services for young people with Attention Deficit/Hyperactivity Disorder (ADHD) in the UK.

**Methods:**

A systematic search of MEDLINE, PsycINFO, Embase, Scopus, ASSIA, and grey literature sources was conducted on 3 June 2025. Eligible studies were qualitative or mixed-method, published from 2014 onwards and examined the transition experiences of young people with ADHD in the UK. Studies were excluded if they were purely quantitative, non-UK, or pre-2014. Methodological quality was appraised using the Critical Appraisal Skills Programme checklist. The data was synthesised using thematic analysis and narrative synthesis.

**Results:**

Nine studies were included: seven peer-reviewed papers and two grey literature reports. Across 224 participants (90 young people, 82 family members, 52 clinicians), three overarching themes were identified: (1) communication as a determinant of transition experience, (2) negotiating discontinuity and change and (3) interdependence of individual and systemic factors.

**Conclusion:**

Transition to adult services for young people with ADHD is commonly experienced as fragmented, poorly coordinated and psychologically distressing. The findings highlight the need for developmentally informed, person-centred transition protocols that recognise transition as an active process rather than a discrete administrative event. Future research should prioritise disengaged young people and investigate long-term trajectories to inform evidence-based policy and service development.

